# Marked Differences in Lower-Limb Muscle Strength and Motor Performance Between Japanese and Chinese Children Aged 9–12: A Cross-National Study

**DOI:** 10.3390/sports13080271

**Published:** 2025-08-15

**Authors:** Kun Niu, Kaoru Tsuyama

**Affiliations:** Graduate School of Health and Sport Science, Nippon Sport Science University, 7-1-1 Fukasawa, Setagaya-ku, Tokyo 158-8508, Japan; tsuyamak@nittai.ac.jp

**Keywords:** hip adductor strength, hip abductor strength, toe grip strength, agility, vigorous-intensity physical activity

## Abstract

Background: This study includes an investigation of lower-limb muscle strength and physical activity among children in Japan and China, with the aim of promoting children’s health. Methods: A total of 564 children (300 boys, 264 girls) aged 9–12 years from public primary schools in Tokyo, Japan, and Jiangxi Province, China, were included. Height, body weight (BW), hip adductor and abductor strength, and toe grip strength were measured. The side-step test (SST) and timed-up-and-go test (TUGT) were performed. Exercise habits were assessed via a questionnaire. Results: Japanese students produced significantly higher values than Chinese students in SST (23.3–37.1%) and TUGT (6.6–8.0%), except among 11-year-old girls. Japanese boys aged 10–11 and girls aged 10–12 had significantly greater hip adductor strength/BW and toe grip strength/BW. Japanese students also showed significantly higher hip abductor strength/BW at all ages. Additionally, the proportion of children engaging in vigorous-intensity physical activity (VPA) was significantly higher in Japanese boys aged 10–12 and girls aged 9–11 than in their Chinese counterparts. Conclusions: Chinese students showed diminished lower-limb strength and agility compared to Japanese students. These findings highlight the importance of promoting outdoor play, particularly VPA, to improve children’s physical fitness and health, especially in China.

## 1. Introduction

Physical inactivity among children is a serious global issue, including in Japan and China. This problem has garnered increasing global attention and is evoking significant concerns about child health [1,2,3].

The World Health Organization (WHO) recommends the following physical activity guidelines for children and adolescents: (1) engagement in moderate-to-vigorous intensity physical activity (MVPA), primarily aerobic, for at least 60 min daily; and (2) participation in vigorous-intensity physical activity (VPA), as well as muscle- and bone-strengthening activities, at least three days per week [4]. However, despite the existence of these guidelines, many children globally fail to achieve the recommended physical activity levels proposed by the WHO [1,2]. Guthold et al. [1] (2020) reported that approximately 80% of school-aged children worldwide do not engage in sufficient physical activity, with this prevalence increasing to 85% among Asian populations. Insufficient physical activity among children is associated with declines in physical fitness, increased rates of childhood obesity, worse eyesight, and mental health issues [5,6].

While physical inactivity is a problem in both Japan and China, it stems from different factors in each country. In China, rapid economic growth in recent years has been linked to various child health issues, including obesity, physical inactivity, and declining eyesight [7]. Due to safety concerns, many Chinese parents rely on private vehicles for transportation, which further reduces children’s physical activity levels [8]. Approximately 170 million children and adolescents in China were classified as insufficiently physically active [9]. To address this issue, the Chinese government has implemented the Healthy China 2030 initiative, which aims to ensure all children engage in at least 60 min of daily physical activity [10]. In Japan, physical fitness assessments among children have been conducted via the Ministry of Education, Culture, Sports, Science and Technology’s physical fitness tests since 1964. Although children’s physical fitness peaked around 1985, it has gradually declined thereafter and remains low to this day [11]. In addition, “Falls” were the most common cause of injuries among children aged 6–12 years, accounting for 43.49% of injuries [12]. Additionally, 30.1% of these children sustained injuries to the face or head during falls, highlighting a lack of proficiency in adopting effective and protective postures when falling among many children [13]. Lower-limb muscle strength and agility are crucial in child development and the prevention of injuries.

Physical activity habits formed in childhood often continue into adulthood [14] and have been shown to influence the maintenance of long-term health [15]. In particular, lower-limb muscle strength is essential for performing basic daily movements such as walking, sitting, standing from a chair, and stair climbing. Insufficient lower-limb muscle strength impairs stability during these activities, increasing the risk of falls and injuries [16]. Specifically, hip adductor strength (HADS), hip abductor strength (HABS), and toe grip strength (TGS) have critical roles in balance control [17,18]. Moreover, TGS was reported to influence running and jumping performance [19]. Therefore, adequate development of lower-limb muscle strength is a critical factor for maintaining children’s overall health.

Although Japan and China are geographically and culturally close, they differ significantly in social environments as well as in the policies and content of physical education [2,20]. However, no study has examined the physical fitness and exercise habits of Japanese and Chinese children from the perspective of lower-limb muscle strength. A cross-national comparison can elucidate the influence of sociocultural contexts on children’s physical development and offer important academic and practical insights.

The purpose of this study was to investigate lower-limb muscle strength and physical activity among children aged 9–12 years in Japan and China, with the goals of identifying cross-national differences and promoting children’s health.

## 2. Materials and Methods

### 2.1. Participants

Participants in this study were 564 children aged 9–12 years enrolled in public primary schools in Japan (Tokyo) and China (Jiangxi Province). The distribution of participants by country, age, and sex was as follows: Japan (9-year-old children: 14 boys, 12 girls; 10-year-old children: 40 boys, 45 girls; 11-year-old children: 41 boys, 34 girls; 12-year-old children: 34 boys, 29 girls) and China (9-year-old children: 34 boys, 36 girls; 10-year-old children: 53 boys, 49 girls; 11-year-old children: 67 boys, 48 girls; 12-year-old children: 17 boys, 11 girls). Table 1 shows participants’ height, body weight (BW), and body mass index (BMI).

The consent procedure involved first thoroughly explaining the purpose and content of the study to the principals and classroom teachers at the target schools and obtaining approval from the principals to conduct the study. Subsequently, written explanations regarding the purpose and content of the study were provided to the participating children and their parents or guardians, and informed consent was obtained from all participants. This study was approved by the Ethics Committee of Nippon Sport Science University (approval numbers: 023-H089 and 023-H153).

### 2.2. Measurements

The measured items included height, BW, HADS, HABS, TGS, the side-step test (SST), and the timed-up-and-go test (TUGT).

**Figure 1:**
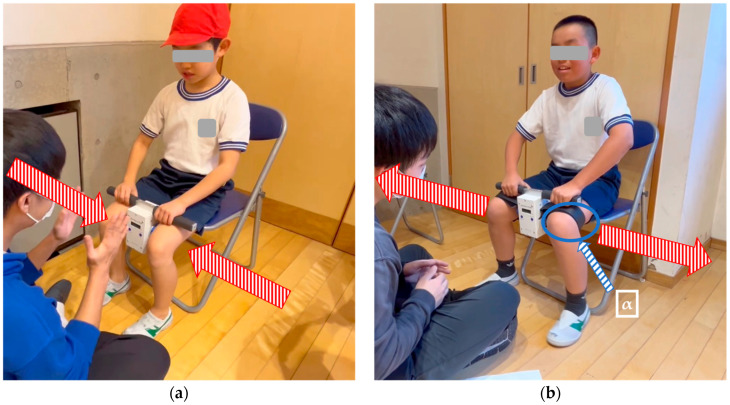
Measurement methods of hip adduction strength (**a**) and hip abduction strength (**b**). Note: (**a**) The red arrow with vertical lines indicates the direction of the applied inward force; (**b**) “**α**” (blue arrow with diagonal lines) indicates the strap used to secure the participant’s leg. The red arrow with vertical lines indicates the direction of the applied outward force.

HADS and HABS were measured simultaneously on both the right and left sides using a hip adduction/abduction dynamometer (T.K.K.3367b, Takei Ltd., Niigata, Japan). Participants were seated with hip and knee joints flexed at 90°. For the HADS measurement, participants applied adduction force by pressing medially against a dynamometer placed between their knees (Figure 1). For the HABS measurement, a strap was wrapped around the middle of the thighs, and participants applied abduction force by pushing laterally against its resistance (Figure 1) [21].

**Figure 2:**
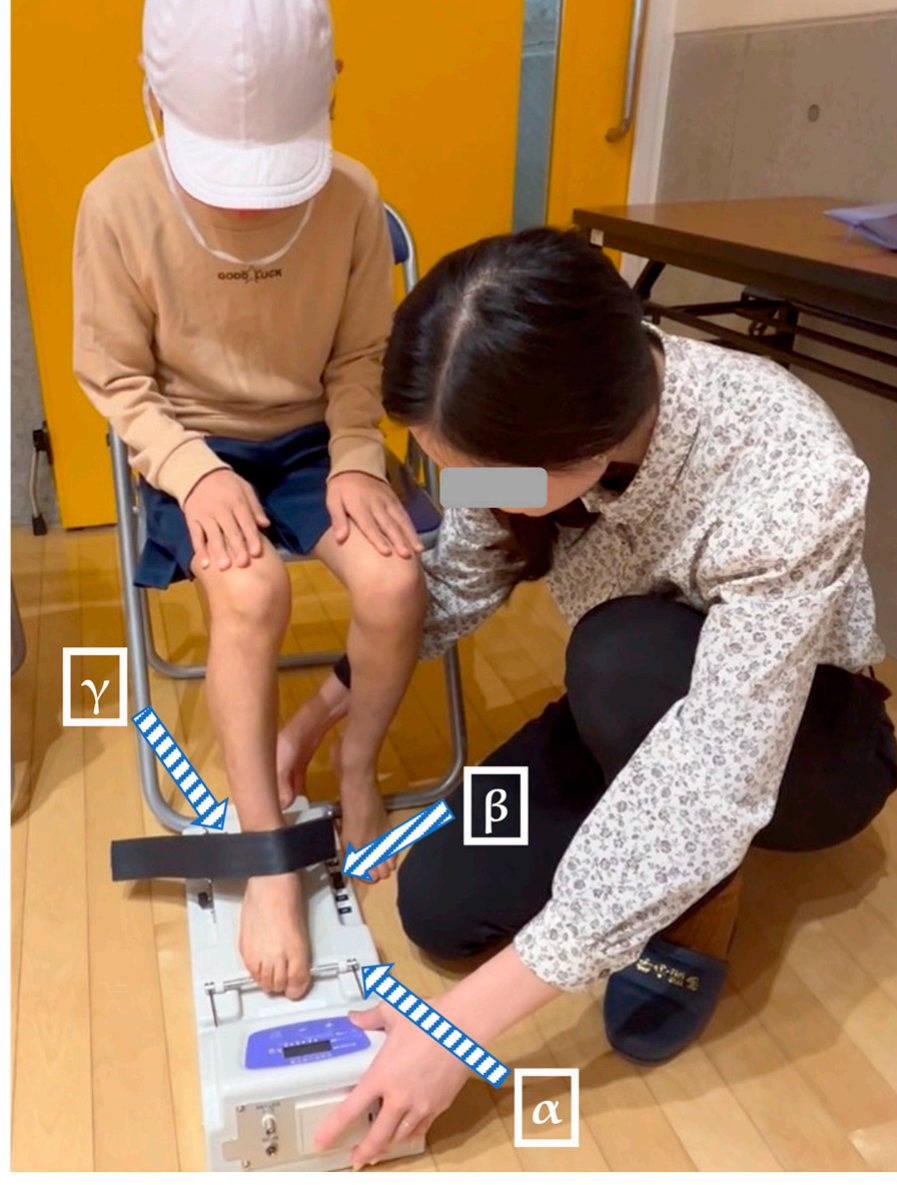
Measurement methods of toe grip strength. Note: “**α**” indicates the measurement bar, “**β**” indicates the adjustment lever, and “**γ**” indicates the fixation strap.

TGS was assessed using a toe grip strength-measuring instrument (T.K.K.3361, Takei Ltd., Niigata, Japan). Participants were seated with their knees and ankles flexed to 90°, and hands resting on their thighs. The measurement was performed using the dominant foot (the foot typically used to kick a ball). The position of the heel was adjusted using a lever, and the first metatarsophalangeal joint was aligned with the measurement bar (Figure 2) [22].

The SST was performed using two parallel lines drawn at intervals of 100 cm from a central line, following established procedures [23]. Participants stood on the central line and, upon a start signal, stepped laterally to touch or cross the line to their right, returned to the center line, and then stepped laterally to touch or cross the line to their left. This movement was repeated as rapidly as possible for 20 s. One time was counted for each line they passed. Jumping was prohibited.

The TUGT was conducted as follows [24]. The participant sat in an adjustable-height chair, with their hips and knees flexed to 90° and feet flat on the floor. “Ready, go” was the signal to initiate movement. On the “go” cue, the participant stood up, walked 3 m, turned around a mark on the floor, walked back, and sat down. The time in seconds was recorded from the “go” cue to when the participant sat down in the chair. Running was prohibited, and the test was repeated if the participant violated this rule.

All tests were performed twice with maximal effort, and the mean of the two values was used for statistical analysis. Participants wore appropriate clothing for physical activity during the measurements. The findings of previous studies reported a positive correlation between BW and lower-limb muscle strength, including knee extensor strength and TGS [19]. Based on our own research (unpublished data), we also confirmed significant positive correlations between BW and both HADS (*r* = 0.613, *p* < 0.001, *n* = 41) and HABS (*r* = 0.596, *p* < 0.001, *n* = 41) in 6th-grade elementary school students. Therefore, to minimize the influence of BW on muscular strength, all strength values were normalized by BW.

Participants’ physical activity levels were assessed using the internationally recognized Health Behaviour in School-aged Children questionnaire recommended by the WHO [25]. Specifically, the questionnaire assessed the following: (1) how many days during the past week participants engaged in at least 60 min of physical activity that increased their heart rate and breathing frequency (MVPA, METs ≥ 3.0) [25] and (2) how many hours per week participants usually spent exercising during their free time, causing them to get out of breath or sweat (VPA, METs > 6.0) [26].

### 2.3. Statistical Analysis

Data are expressed as mean ± standard deviation. Non-parametric data are presented as median (range). Normality of the data was assessed using the Shapiro–Wilk test for sample sizes below 50 and the Kolmogorov–Smirnov test for sample sizes of 50 or greater.

For comparisons of muscle strength and motor performance, an independent t-test was conducted for variables with a normal distribution, whereas the Mann–Whitney U test was applied to those without a normal distribution. The Chi-Square test compared the proportions of children engaging in MVPA and VPA. For MVPA, the proportion of children who engaged in at least 60 min of MVPA daily was compared between the Japanese and Chinese students. For VPA, participants were categorized into two groups based on weekly duration: those who were engaged ≥ 2 h per week and those who were engaged < 2 h. The use of the 2 h threshold was exploratory, as the WHO recommends engaging in vigorous physical activity at least three days per week but does not provide a specific duration.

All statistical analyses were performed using SPSS Statistics for macOS, Version 29.0.2.0 (IBM Corp., Armonk, NY, USA), and statistical significance was set at 5%.

## 3. Results

The HADS/BW and TGS/BW of the Japanese students were significantly higher than those of the Chinese students among boys aged 10–11 years and girls aged 10–12 years. Furthermore, the HABS/BW of the Japanese students was significantly higher than that of the Chinese students across all age groups in both sexes (Table 2). Although all measurement procedures were standardized, the chairs used during the lower-limb strength tests were not height-adjustable, which may have slightly affected the test posture of some participants.

Significant differences in SST and TUGT were observed between Japanese and Chinese students across all age groups and both sexes, except for the 11-year-old girls. In the results derived from the Japanese students compared to those from the Chinese students, the SST and TUGT values were 23.3–37.1% and 6.6–8.0% higher, respectively (Table 3).

No significant differences were observed in the proportion of Japanese and Chinese students engaging in MVPA. However, the proportion of children engaging in ≥2 h of VPA per week among Japanese students was significantly higher than that of Chinese students, particularly among boys aged 10–12 years and girls aged 9–11 years. Importantly, among girls aged 9–10 years, the proportion of students engaging in ≥2 h of VPA per week among the Japanese students was more than four times that of the Chinese students (Table 4).

## 4. Discussion

This study includes a comparison of lower-limb muscle strength, motor performance, and participation in physical activity between Japanese and Chinese primary school children. Chinese children demonstrated significantly lower values for muscle strength (HADS/BW, HABS/BW, and TGS/BW), motor performance (SST and TUGT), and a lower proportion of children engaging in ≥2 h of VPA per week.

Yang et al. [27] (2019) compared VO_2max_ between Japanese and Chinese children aged 7–18 years (n = 9025) and reported that the cardiorespiratory fitness of Chinese children was significantly lower than that of Japanese children and that physical activity among Chinese children was insufficient. Li et al. [28] (2020) reported that Chinese children’s agility, assessed by SST, and aerobic fitness, assessed by a 20 m shuttle run, were significantly lower than those of Japanese children. Consistent with their findings, the present study also revealed that Chinese children showed significantly lower SST.

In a longitudinal study [29] conducted in Nagano, SST values among children in grades 4–6 (approximately 10–12 years old) ranged from 39.9 ± 5.9 to 47.6 ± 7.4 repetitions. Similarly, a study [23] from Hokkaido reported that SST values ranged from 39.8 ± 4.9 to 41.4 ± 8.1 repetitions among fifth-grade students. These values are comparable to those observed in the present study on Japanese children aged 9–12 years in Tokyo (SST values ranged from 33.3 ± 4.7 to 43.6 ± 5.7 repetitions). In contrast, Chinese children in the same age group demonstrated markedly lower SST performance, with values from 27.0 ± 3.3 to 31.8 ± 4.9 repetitions. SST is an important assessment of agility [30], and is closely related to neurological development in children, significantly improving during early to middle childhood [31]. The age of 9–12 years is often termed the “golden age” and represents a critical period for neurological development [32]. However, Chinese children showed significantly lower SST values, as well as lower values of lower-limb muscle strength (HADS/BW and HABS/BW). These findings suggest that Chinese children are not sufficiently engaged in physical activities that promote agility during early to middle childhood.

Compared with China, the Japanese government emphasizes developing fundamental motor abilities, such as balance, agility, and coordination, particularly in early primary education [20]. In contrast, the Chinese government understates curricula designed to enhance fundamental motor skills [20]. This curricular focus in Chinese school physical education may negatively impact neurological development, including agility.

In this study, the TGS/BW of Chinese children was significantly lower than that of Japanese children in some grades. TGS has been positively correlated with physical performance indicators such as the 50 m sprint (running ability), standing long jump (jumping ability), and the functional reach test (balance ability) [18,19]. Tsuyama [19] reported TGS/BW values among third-grade elementary school students (approximately 9 years old) in Japan, with boys showing 0.37 ± 0.08 kg/kg and girls 0.38 ± 0.10 kg/kg. The present study found higher TGS/BW values for 9-year-old Japanese children than those reported by Tsuyama and extremely low values for Chinese children.

These findings suggest that Chinese children’s engagement in daily activities involving running and jumping is lower than that of Japanese children. This interpretation is further supported by the results of the TUGT, which is related to balance, coordination, and cognitive function [33,34]. In the present study, Chinese children showed a tendency to expend significantly more time in the TUGT than Japanese children.

Marchese et al. [35] reported that the median (range) TUGT values for children aged 10–12 years in the United States ranged from 3.48 (2.68–4.52) to 4.40 (3.27–5.29) seconds. In the present study, Japanese children aged 9–12 years recorded TUGT values from 4.30 ± 0.42 to 4.53 ± 0.48 s, whereas Chinese children in the same age group showed slightly slower performance, with values from 4.62 ± 0.37 to 4.86 ± 0.34 s.

Comparisons of physical activity habits between the two countries revealed no significant differences in participation in MVPA. However, the proportion of Chinese children engaging in at least 2 h of VPA per week was 58.3–81.0% lower than that of Japanese children.

These findings suggest that differences in lower-limb muscle strength (HADS/BW, HABS/BW, and TGS/BW) and motor performance (SST and TUGT) between Japanese and Chinese children may be attributable to a lower proportion of Chinese children engaging in at least 2 h of VPA per week.

In China, most children spend a lot of time studying outside of school, which limits their opportunities for physical activity after school. In contrast, Japanese primary school students reportedly have more free time after school, which helps to enable greater participation in sports and outdoor play [2,36]. Moreover, participation in after-school sports clubs differs markedly between the two countries. Approximately 60–66% of Japanese children participate in such activities; however, Chinese children show a much lower proportion of participation, at less than 20% [2].

Reis et al. [37] reported that weekly participation in approximately 2 h of physical activity sessions primarily focused on VPA among children aged 6–11 years was associated with improvements in agility, cardiorespiratory fitness, and lower-limb muscle strength. They also reported that increasing physical education classes is insufficient and additional opportunities for physical activity outside of school are necessary, such as outdoor play and participation in sports clubs, to promote VPA. Benavente-Marín et al. [38] reported that participation in more than 3 h per week of sports activities outside school significantly enhances children’s VPA levels. Therefore, increasing opportunities for physical activity outside of school may enhance children’s participation in VPA and support improvements in their physical fitness.

It has been reported that physical activity habits established during childhood tend to track into later life, with regular physical activity from early childhood through school age positively influencing health outcomes not only during childhood but also into adulthood [14]. Therefore, it is essential that children actively participate in outdoor physical activities involving running and jumping, both within and outside school settings. This is especially important in China, but also relevant in Japan. Enhancing lower-limb muscle strength and agility through these activities is essential to promoting and maintaining children’s long-term health.

The primary focus of this study was lower-limb muscle strength and motor performance, without accounting for other possible influencing factors such as nutrition and socioeconomic background. In addition, its cross-sectional design limits the ability to draw causal inferences. Future studies should take a longitudinal approach and incorporate a broader range of variables to deepen the understanding of children’s physical development.

## 5. Conclusions

Compared with Japanese children, Chinese children showed significantly lower performance in HABS and SST, which reflect agility, and the proportion of children engaging in VPA was also markedly lower in China. To our knowledge, this is one of the first studies to conduct a cross-national comparison of lower-limb muscle strength, agility-related motor performance, and physical activity levels between Japanese and Chinese primary school children. These findings suggest the importance of actively incorporating VPA, particularly through outdoor play, into children’s daily lives. Although these findings are particularly relevant to Chinese students, they also apply to Japanese students.

As this was a cross-sectional study, causal relationships cannot be determined, and unmeasured factors such as maturation and socioeconomic background may have influenced the results. Future longitudinal studies should address these variables. In addition, we recommend that physical education teachers and related professionals increase opportunities for VPA and agility training, both in school and extracurricular settings. These findings also offer practical guidance for national-level policies aimed at improving children’s long-term physical health.

## Figures and Tables

**Table 1:** Physical characteristics of the participants.

Variable	Age (Years)	Boys			*p*	Effect Size (Cohen’s d/r)	Girls			*p*	Effect Size (Cohen’s d/r)
		nC, nJ	China	Japan			nC, nJ	China	Japan		
Height (cm)	9	34, 14	136.5 ± 7.6	134.7 (132.4–141.5)	1.000 ^‡^	0.000	36, 12	135.5 ± 6.2	136.5 ± 7.3	0.643 ^†^	0.155
	10	53, 40	141.1 ± 6.3	139.7 ± 7.0	0.312 ^†^	−0.213	49, 45	144.2 ± 7.0	139.9 ± 7.2	**0.004 ^†^**	−0.611
	11	67, 41	147.5 ± 7.8	145.4 ± 6.2	0.154 ^†^	−0.284	48, 34	150.3 ± 7.3	147.2 ± 5.6	**0.041 ^†^**	−0.465
	12	17, 34	151.8 ± 6.0	154.9 ± 7.4	0.143 ^†^	0.442	11, 29	153.4 ± 7.1	153.5 ± 4.8	0.936 ^†^	0.028
Body Weight (kg)	9	34, 14	33.1 (27.7–42.7)	31.7 (28.4–34.3)	0.856 ^‡^	−0.026	36, 12	27.3 (25.9–30.7)	31.1 ± 6.0	0.225 ^‡^	0.175
	10	53, 40	33.2 (30.1–38.1)	34.4 (29.4–38.8)	0.923 ^‡^	−0.001	49, 45	35.6 (31.4–39.3)	32.8 (27.6–39.8)	0.193 ^‡^	−0.134
	11	67, 41	38.4 (34.6–45.6)	36.8 (34.2–41.4)	0.268 ^‡^	−0.107	48, 34	38.4 (34.0–45.7)	38.1 ± 6.0	0.273 ^‡^	−0.121
	12	17, 34	40.6 (38.8–43.7)	45.2 (40.8–53.7)	0.055 ^‡^	0.269	11, 29	42.9 ± 6.2	42.3 ± 5.8	0.842 ^†^	0.071
BMI (kg/m^2^)	9	34, 14	17.6 (15.9–21.3)	17.1 (16.2–18.1)	0.634 ^‡^	−0.069	36, 12	15.3 (14.4–16.1)	16.5 ± 1.6	**0.036 ^‡^**	0.302
	10	53, 40	16.8 (16.0–18.0)	17.2 (15.9–19.0)	0.475 ^‡^	0.074	49, 45	17.0 (15.2–18.4)	16.6 (14.9–20.0)	0.835 ^‡^	−0.021
	11	67, 41	17.4 (16.4–20.6)	17.4 (16.3–19.3)	0.660 ^‡^	−0.042	48, 34	16.9 (16.0–19.7)	17.5 ± 2.1	0.932 ^‡^	0.009
	12	17, 34	18.4 ± 2.3	19.2 (17.5–21.0)	0.134 ^‡^	0.210	11, 29	17.8 ± 2.5	17.9 ± 2.2	0.903 ^†^	0.044

Note: Data are presented as means ± standard deviations or medians (ranges); nC = number of participants in China, nJ = number of participants in Japan; ^†^ = analyzed using independent *t*-test; ^‡^ = analyzed using Mann–Whitney U test; BMI = body mass index. bold: significant *p* values.

**Table 2:** Comparison of lower-limb muscle strength relative to body weight between Japanese and Chinese participants by age in boys and girls.

Variable	Age (Years)	Boys			*p*	Effect Size (Cohen’s d/r)	Girls			*p*	Effect Size (Cohen’s d/r)
		nC, nJ	China	Japan			nC, nJ	China	Japan		
HADS/BW (kg/kg)	9	34, 14	0.34 ± 0.10	0.37 ± 0.14	0.382 ^†^	0.280	36, 12	0.33 ± 0.09	0.37 ± 0.07	0.175 ^†^	0.459
	10	53, 40	0.37 ± 0.09	0.41 ± 0.10	**0.025 ^†^**	0.477	49, 45	0.33 ± 0.08	0.39 ± 0.10	**<0.001 ^†^**	0.719
	11	67, 41	0.36 ± 0.10	0.43 ± 0.10	**0.003 ^†^**	0.604	48, 34	0.34 (0.31–0.41)	0.46 (0.39–0.50)	**<0.001 ^‡^**	0.543
	12	17, 34	0.40 ± 0.12	0.43 (0.39–0.49)	0.197 ^‡^	0.180	11, 29	0.34 ± 0.06	0.43 ± 0.09	**0.002 ^†^**	1.174
HABS/BW (kg/kg)	9	34, 14	0.23 (0.17–0.31)	0.46 (0.40–0.62)	**<0.001 ^‡^**	0.697	36, 12	0.22 ± 0.07	0.53 ± 0.12	**<0.001 ^†^**	3.754
	10	53, 40	0.28 ± 0.08	0.56 ± 0.12	**<0.001 ^†^**	2.778	49, 45	0.22 (0.18–0.31)	0.56 ± 0.16	**<0.001 ^‡^**	0.781
	11	67, 41	0.23 (0.19–0.29)	0.59 ± 0.14	**<0.001 ^‡^**	0.808	48, 34	0.23 (0.16–0.28)	0.59 ± 0.12	**<0.001 ^‡^**	0.829
	12	17, 34	0.26 ± 0.09	0.55 ± 0.12	**<0.001 ^†^**	2.675	11, 29	0.21 ± 0.07	0.54 ± 0.11	**<0.001 ^†^**	3.338
TGS/BW (kg/kg)	9	34, 14	0.34 (0.28–0.42)	0.42 ± 0.12	0.057 ^‡^	0.275	36, 12	0.35 ± 0.08	0.40 ± 0.11	0.105 ^†^	0.551
	10	53, 40	0.35 ± 0.12	0.40 ± 0.10	**0.043 ^†^**	0.429	49, 45	0.33 ± 0.10	0.38 ± 0.11	**0.017 ^†^**	0.501
	11	67, 41	0.32 ± 0.11	0.40 ± 0.10	**<0.001 ^†^**	0.723	48, 34	0.33 ± 0.09	0.43 (0.38–0.51)	**<0.001 ^‡^**	0.548
	12	17, 34	0.38 ± 0.12	0.41 ± 0.10	0.251 ^†^	0.345	11, 29	0.37 ± 0.08	0.44 (0.39–0.51)	**0.020 ^‡^**	0.366

Note: Data are presented as means ± standard deviations or medians (ranges); nC = number of participants in China, nJ = number of participants in Japan; ^†^ = analyzed using independent *t*-test; ^‡^ = analyzed using Mann–Whitney U test; HADS/BW = hip adduction strength relative to body weight; HABS/BW = hip abduction strength relative to body weight; TGS/BW = toe grip strength relative to body weight. bold: significant *p* values.

**Table 3:** Comparison of motor performance between Japanese and Chinese participants by age in boys and girls.

Variable	Age (Years)	Boys			*p*	Effect Size (Cohen’s d/r)	Girls			*p*	Effect Size (Cohen’s d/r)
		nC, nJ	China	Japan			nC, nJ	China	Japan		
SST (repetitions)	9	34, 14	28.5 ± 3.4	38.3 ± 7.2	**<0.001 ^†^**	2.051	36, 12	27.0 ± 3.3	33.3 ± 4.7	**<0.001 ^†^**	1.717
	10	53, 40	29.6 ± 4.7	38.5 ± 5.4	**<0.001 ^†^**	1.759	49, 45	28.0 (26.0–30.5)	36.2 ± 5.4	**<0.001 ^‡^**	0.680
	11	67, 41	30.8 ± 4.6	41.6 ± 6.3	**<0.001 ^†^**	2.044	48, 34	29.8 ± 4.1	39.9 ± 4.6	**<0.001 ^†^**	2.343
	12	17, 34	31.8 ± 4.9	43.6 ± 5.7	**<0.001 ^†^**	2.194	11, 29	31.2 ± 4.0	40.3 ± 6.5	**<0.001 ^†^**	1.524
TUGT (s)	9	34, 14	4.66 ± 0.41	4.30 ± 0.42	**0.007 ^†^**	−0.889	36, 12	4.82 ± 0.46	4.50 ± 0.43	**0.039 ^†^**	−0.710
	10	53, 40	4.73 (4.42–4.85)	4.38 ± 0.45	**0.002 ^‡^**	−0.326	49, 45	4.79 ± 0.40	4.42 (4.10–4.77)	**0.001 ^‡^**	−0.337
	11	67, 41	4.67 (4.36–4.97)	4.34 (4.11–4.66)	**0.001 ^‡^**	−0.314	48, 34	4.70 ± 0.50	4.53 ± 0.48	0.138 ^†^	−0.336
	12	17, 34	4.62 ± 0.37	4.30 (4.07–4.66)	**0.046 ^‡^**	−0.280	11, 29	4.86 ± 0.34	4.47 ± 0.46	**0.01 4^†^**	−0.911

Note: Data are presented as means ± standard deviations or medians (ranges); nC = number of participants in China, nJ = number of participants in Japan; ^†^ = analyzed using independent *t*-test; ^‡^ = analyzed using Mann–Whitney U test; SST = side-step test; TUGT = timed-up-and-go test. bold: significant *p* values.

**Table 4:** Comparison of physical activity levels between Japanese and Chinese participants by age in boys and girls.

Variable	Age (Years)	Boys			*p*	Effect Size (OR, 95% CI)	Girls			*p*	Effect Size (OR, 95% CI)
		nC, nJ	China	Japan			nC, nJ	China	Japan		
MVPA	9	34, 14	10 (29.4)	4 (28.6)	0.954	0.988 (0.665–1.468)	36, 12	3 (8.3)	3 (25.0)	0.131	1.222 (0.869–1.719)
	10	53, 40	16 (30.2)	7 (17.5)	0.160	0.846 (0.674–1.062)	49, 45	11 (22.4)	8 (17.8)	0.573	0.943 (0.770–1.155)
	11	67, 41	15 (22.4)	10 (24.4)	0.811	1.026 (0.827–1.274)	48, 34	6 (12.5)	3 (8.8)	0.600	0.960 (0.826–1.114)
	12	17, 34	3 (17.6)	6 (17.6)	1.000	1.000 (0.764–1.309)	11, 29	2 (18.2)	2 (6.9)	0.288	0.879 (0.654–1.181)
VPA	9	34, 14	7 (20.6)	6 (42.9)	0.115	1.390 (0.856–2.257)	36, 12	4 (11.1)	7 (58.3)	**<0.001**	2.133 (1.081–4.208)
	10	53, 40	14 (26.4)	29 (72.5)	**<0.001**	2.676 (1.578–4.539)	49, 45	5 (10.2)	21 (46.7)	**<0.001**	1.684 (1.261–2.248)
	11	67, 41	15 (22.4)	22 (53.7)	**<0.001**	1.675 (1.176–2.385)	48, 34	8 (16.7)	14 (41.2)	**0.014**	1.417 (1.041–1.928)
	12	17, 34	3 (17.6)	16 (47.1)	**0.041**	1.556 (1.058–2.288)	11, 29	3 (27.3)	13 (44.8)	0.312	1.318 (0.809–2.148)

Note: Data are expressed as n (%); nC = number of participants in China, nJ = number of participants in Japan; MVPA = proportion of children who engaged in ≥60 min of moderate-to-vigorous physical activity daily; VPA = proportion of children who engaged in ≥2 h of vigorous-intensity physical activity per week. bold: significant *p* values.

## Data Availability

The datasets used in this study are available from the corresponding author upon reasonable request due to privacy and ethical reasons.
